# Data for the synthesis of β-oxopropylcarbamates from propargylic alcohols, secondary amines and CO_2_ catalyzed by a recyclable AgBr/ionic liquid system under ambient pressure

**DOI:** 10.1016/j.dib.2018.08.183

**Published:** 2018-09-05

**Authors:** Dandan Song, Di Li, Xuan Xiao, Cheng Chen, Somboon Chaemchuen, Ye Yuan, Francis Verpoort

**Affiliations:** a State Key Laboratory of Advanced Technology for Materials Synthesis and Processing, Wuhan University of Technology, Wuhan 430070, PR China; b School of Material Science and Engineering, Wuhan University of Technology, Wuhan 430070, PR China; c National Research Tomsk Polytechnic University, Lenin Avenue 30, Tomsk 634050, Russian Federation; d Global Campus Songdo, Ghent University, 119 Songdomunhwa-Ro, Yeonsu-Gu, Incheon, Republic of Korea

## Abstract

Data presented here are related with the research article entitled “Synthesis of ß -oxopropylcarbamates in a recyclable AgBr/ionic liquid catalytic system: An efficient assembly of CO_2_ under ambient pressure” (Song et al., 2018) [1]. In this data article, the general synthetic procedures of ß-oxopropylcarbamates through the three-component reaction of propargylic alcohols, secondary amines and carbon dioxide (CO_2_) catalyzed by a recyclable AgBr/ionic liquid (IL) system under mild pressure are described. Furthermore, the process for recycling the catalysts is supplied as well. Specifically, the investigative data for the temperature, amount of ILs, reaction time as well as the state of silver in the system are also reported. Finally, all the target products are confirmed by ^1^H NMR, ^13^C NMR, and high-resolution mass spectroscopy (HR-MS).

**Specifications table**

**Table:** 

Subject area	*Chemistry*
More specific subject area	*Carbon dioxide utilization*
Type of data	*Figures, tables*
How data was acquired	*A Bruker Avance 500 spectrometer NMR instrument, a Bruker Daltonics microTOF-QII mass spectrometry instrument.*
Data format	*Raw, analyzed.*
Experimental factors	*Ordinary reagents and solvents were commercially available and used without further manipulation.*
Experimental features	*NMR analysis: Bruker Avance 500 spectrometer NMR instrument; HR-MS analysis: a Bruker Daltonics microTOF-QII mass spectrometry instrument.*
Data source location	*Wuhan, China*
Data accessibility	*Data is provided within the article.*
Related research article	*Dandan Song, Di Li, Xuan Xiao, Chen Cheng, Somboon Chaemchuen, Ye Yuan, Francis Verpoort, Synthesis of ß-Oxopropylcarbamates in a Recyclable AgBr/Ionic Liquid Catalytic System: an Efficient Assembly of CO*_*2*_*under Ambient Pressure. J. CO_2_ Util., 27(2018), 217–222.*

**Value of the data**

• The procedures for the synthesis of ß-oxopropylcarbamates and the process for recycling the catalysts could be followed by other researchers.
• The characterization data of various ß-oxopropylcarbamates would be useful for the structural verification in other research.
• The investigative data for the temperature, amount of ILs and reaction time could help to establish the optimal catalytic conditions easily in related research.
• The investigative data of the probable state of Ag in the system would inspire other researchers to explore more possibilities in the mechanism study.

## 1 Data

The general information including the starting chemicals and the characterization instruments are first supplied, especially the structures of the ILs which have been illustrated in Fig. 1. The general synthetic procedures of ß-oxopropylcarbamates and the process for recycling the catalysts are then described in detail. Moreover, the investigative data for the temperature, amount of ILs and reaction time are summarized in Table 1, Table 2, Table 3. Particularly, the spectra for the investigation of probable state of Ag are offered in Fig. 3, Fig. 4. Finally, Fig. 5, Fig. 6, Fig. 7, Fig. 8, Fig. 9, Fig. 10, Fig. 11, Fig. 12, Fig. 13, Fig. 14, Fig. 15, Fig. 16, Fig. 17, Fig. 18, Fig. 19, Fig. 20, Fig. 21, Fig. 22, Fig. 23, Fig. 24, Fig. 25, Fig. 26 shows the ^1^H NMR and ^13^C NMR spectra of all the target compounds.

**Fig. 1:**
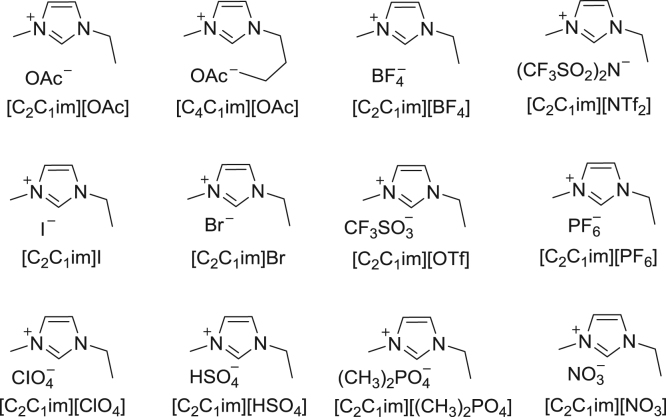
The structures of the applied ionic liquids.

**Fig. 2:**
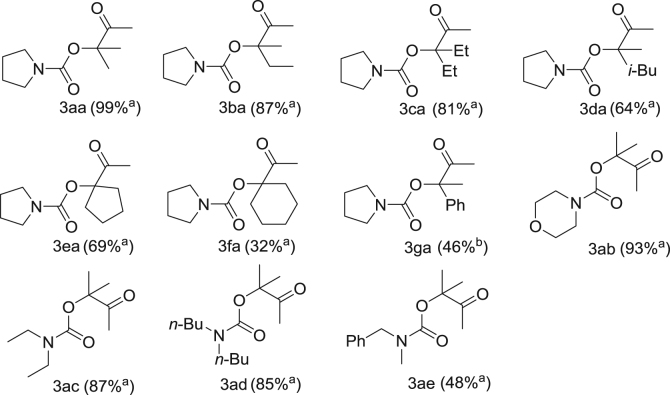
Structure of compounds **3aa-3ae**. ^a^ Reaction conditions: AgBr (0.05 mmol), [C_2_C_1_im][OAc] (1.25 mmol), propargylic alcohols (5 mmol), secondary amines (5 mmol), CO_2_ (0.1 MPa), 45 °C. Yields were determined by ^1^H NMR spectroscopy using 1,3,5-trimethoxybenzene as the internal standard. ^b^ [C_2_C_1_im][OAc] (6.47 mmol).

**Table 1:** Screening of the optimal amount of [C_2_C_1_im][OAc]^a^.

Entry	[C_2_C_1_im][OAc]	%-Yield^b^
1	0.32 mmol	57
2	0.65 mmol	72
3	0.97 mmol	85
4	1.25 mmol	95
5	1.62 mmol	96

a Reaction conditions: AgBr (0.05 mmol), 2-methylbut-3-yn-2-ol (5 mmol), pyrolidine (5 mmol), CO_2_ (0.1 MPa), 45 °C, 8 h.

b Yields were determined by ^1^H NMR spectroscopy using 1,3,5-trimethoxybenzene as the internal standard.

**Table 2:** Screening of the optimal temperature^a^.

Entry	*T*/°C	%-Yield^b^
1	25	16
2	45	95
3	65	94

a Reaction conditions: AgBr (0.05 mmol), [C_2_C_1_im][OAc] (1.25 mmol), 2-methylbut-3-yn-2-ol (5 mmol), pyrolidine (5 mmol), CO_2_ (0.1 MPa), 8 h.

b Yields were determined by ^1^H NMR spectroscopy using 1,3,5-trimethoxybenzene as the internal standard.

**Table 3:** Exploring of the relationship between time and %-yield^a^.

Entry	*t*/h	%-Yield^b^
1	5	70
2	7	93
3	9	99
4	12	> 99

a Reaction conditions: AgBr (0.05 mmol), [C_2_C_1_im][OAc] (1.25 mmol), 2-methylbut-3-yn-2-ol (5 mmol), pyrolidine (5 mmol), CO_2_ (0.1 MPa), 45 °C.

b Yields were determined by ^1^H NMR spectroscopy using 1,3,5-trimethoxybenzene as the internal standard.

**Fig. 3:**
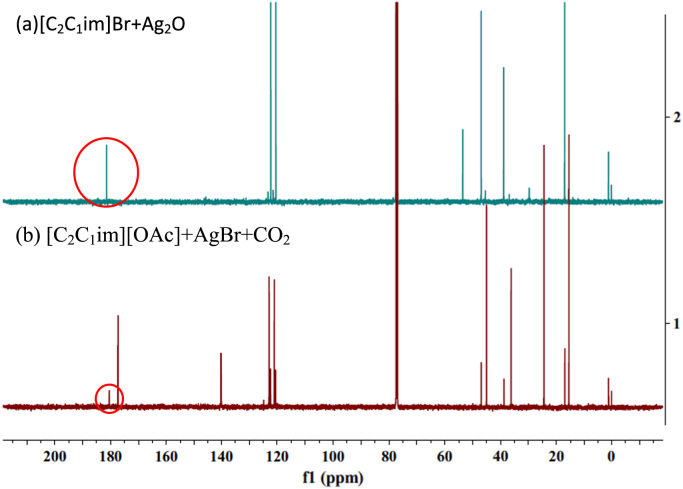
^13^C NMR for detection of the existence of carbene-Ag complex.

**Fig. 4:**
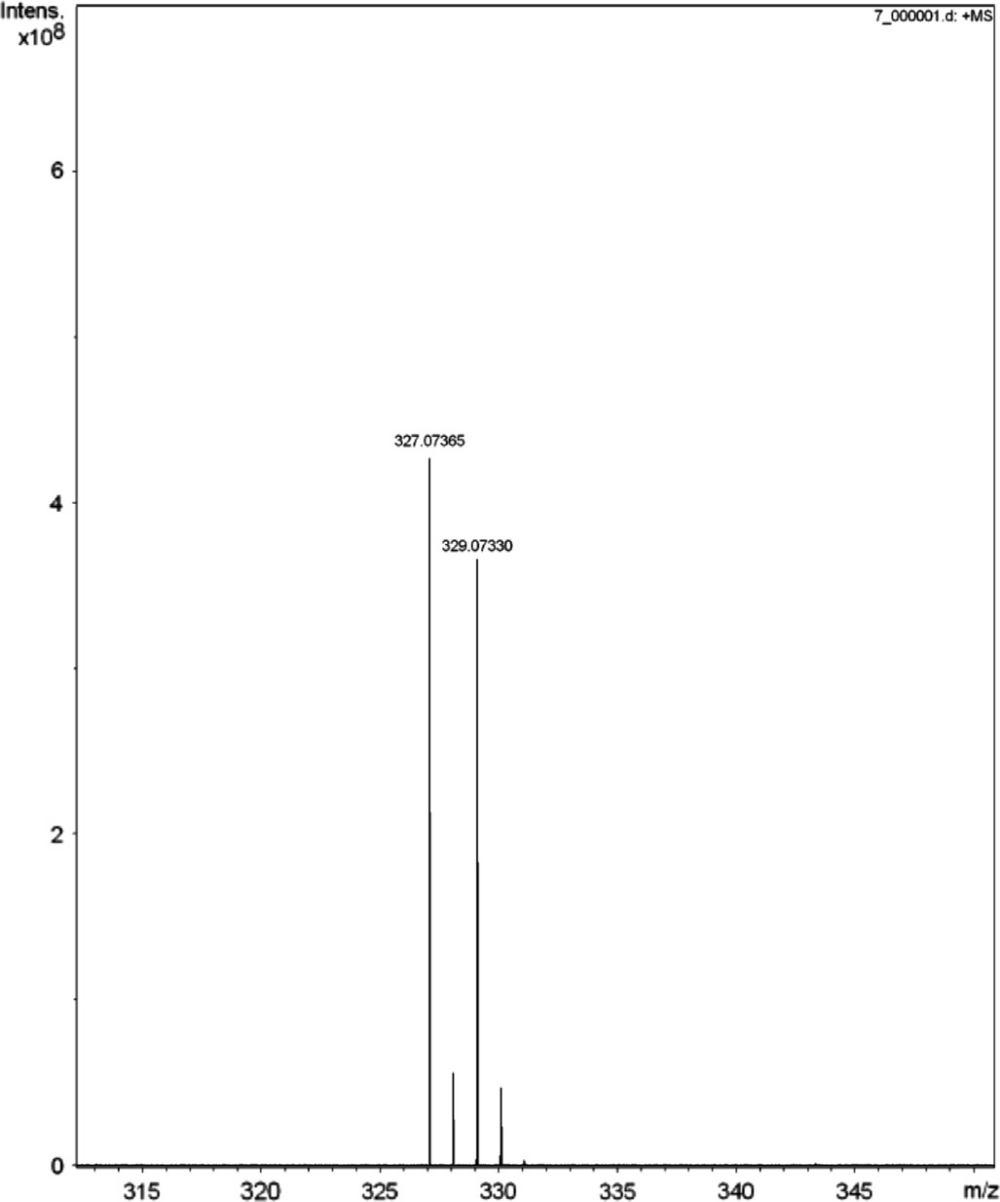
HRMS of N-heterocyclic bis-carbene silver complex.

**Fig. 5:**
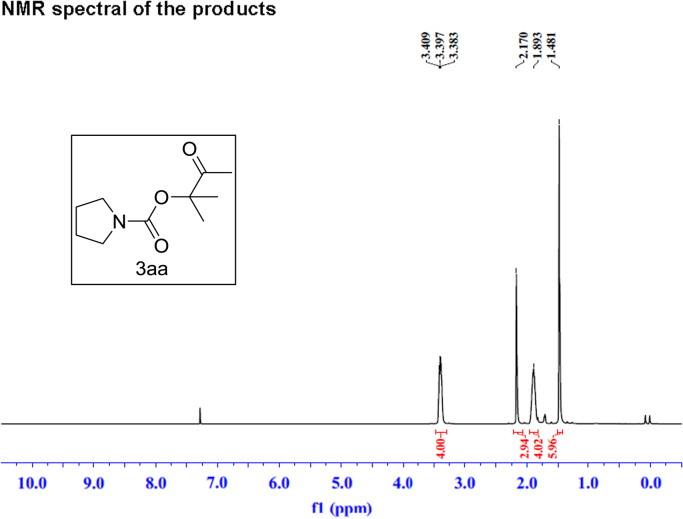
^1^H NMR spectra of **3aa**.

**Fig. 6:**
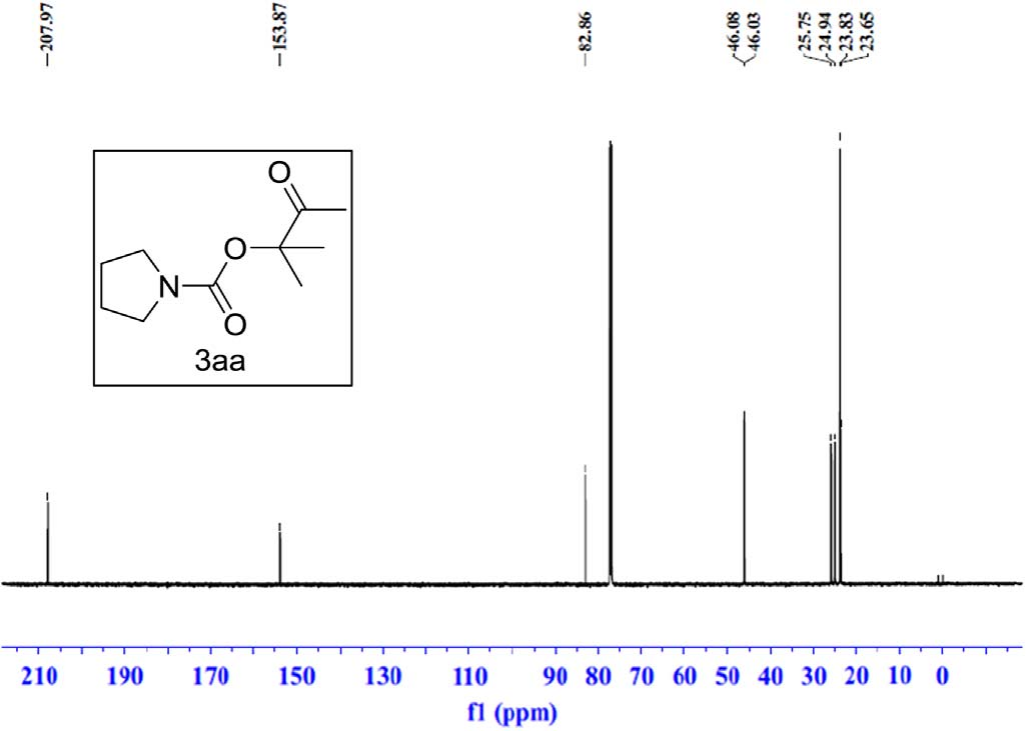
^13^C NMR spectra of **3aa**.

**Fig. 7:**
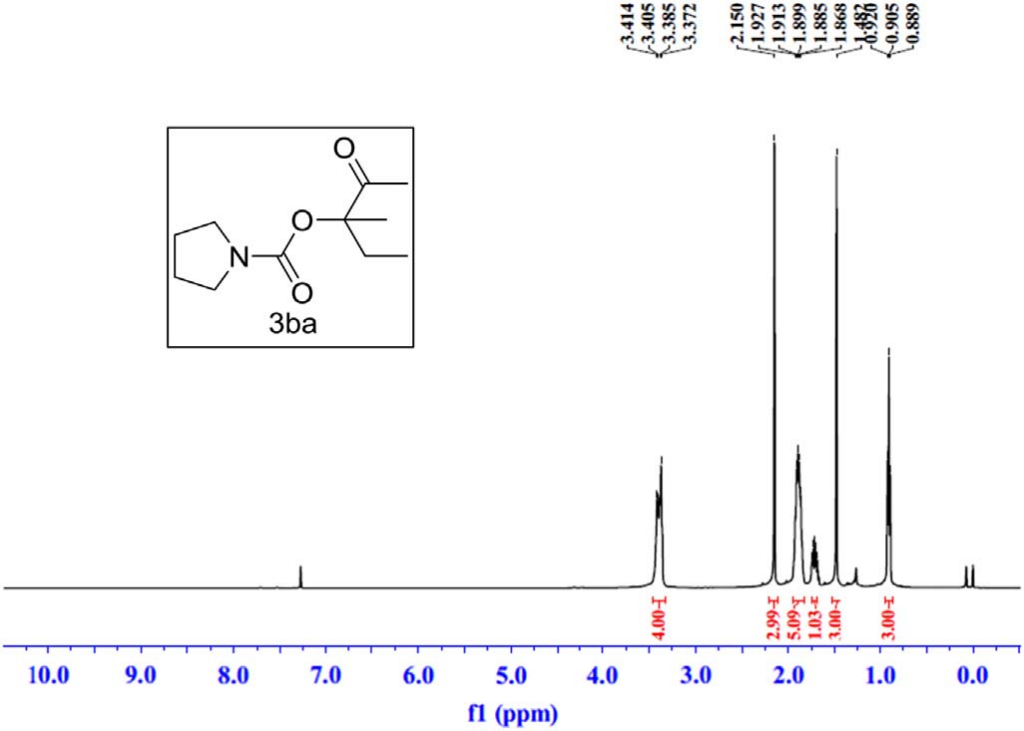
^1^H NMR spectra of **3ba**.

**Fig. 8:**
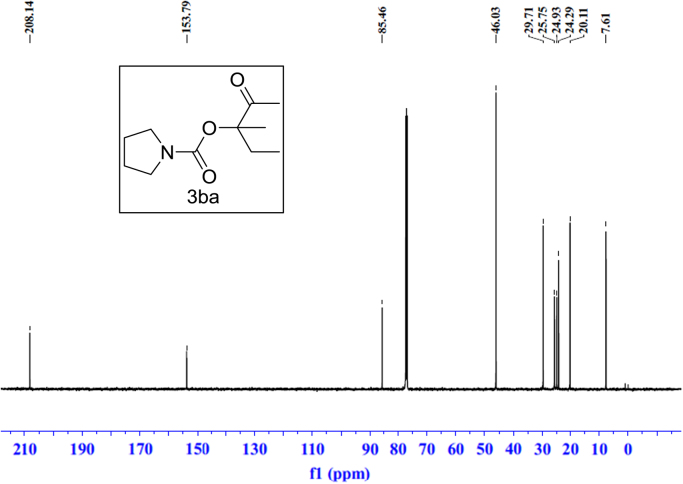
^13^C NMR spectra of **3ba**.

**Fig. 9:**
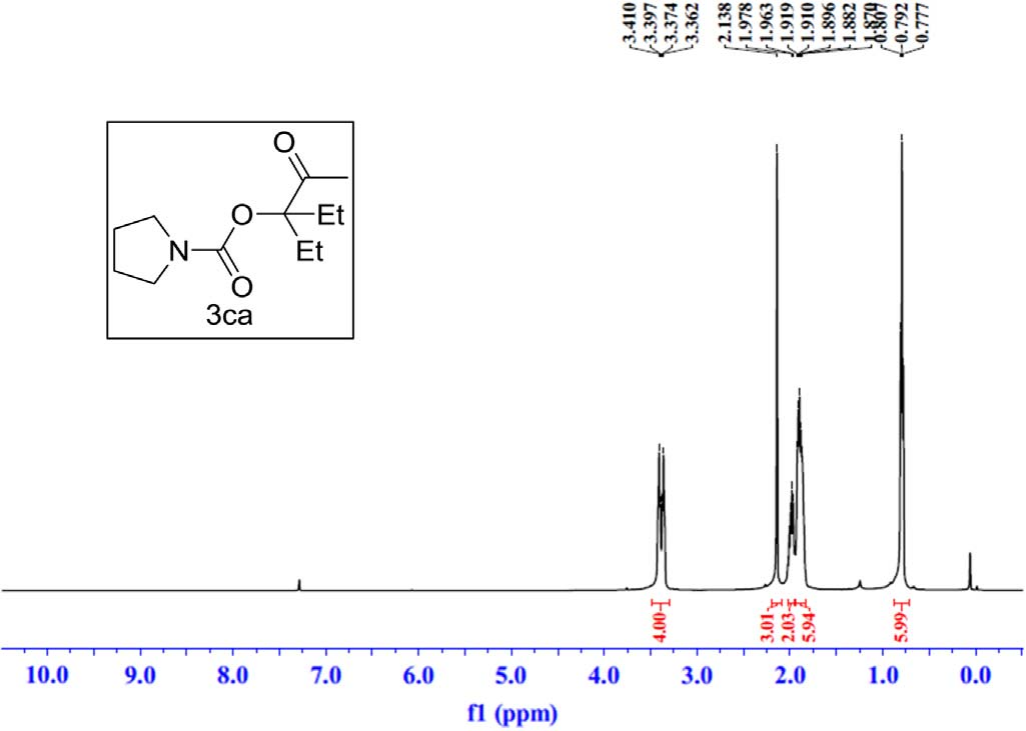
^1^H NMR spectra of **3ca**.

**Fig. 10:**
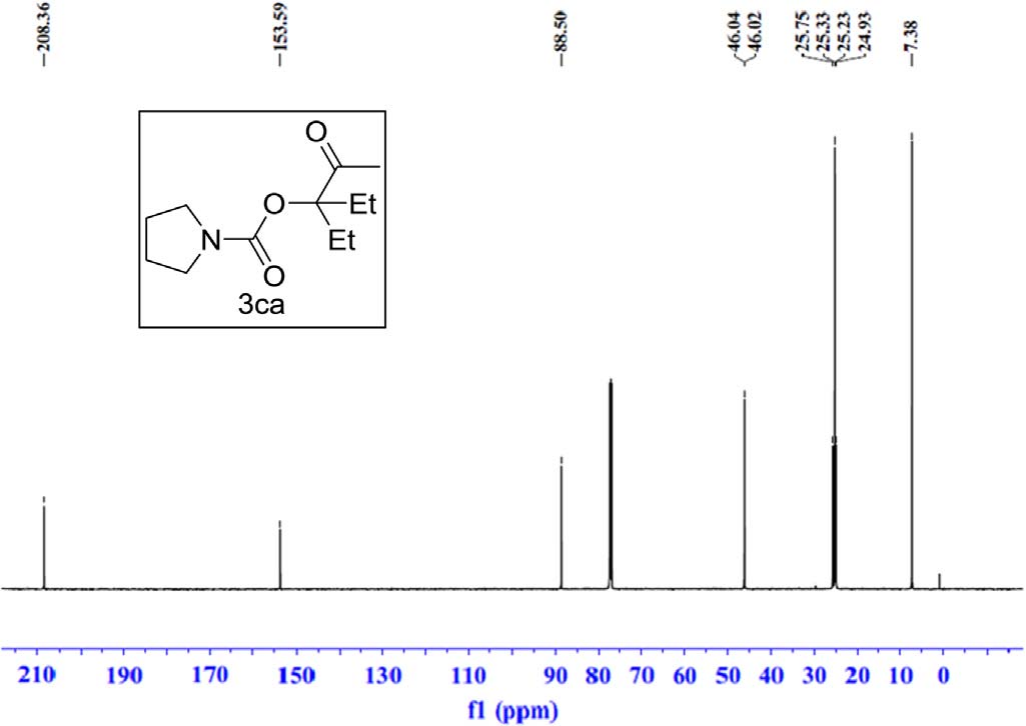
^13^C NMR spectra of **3ca**.

**Fig. 11:**
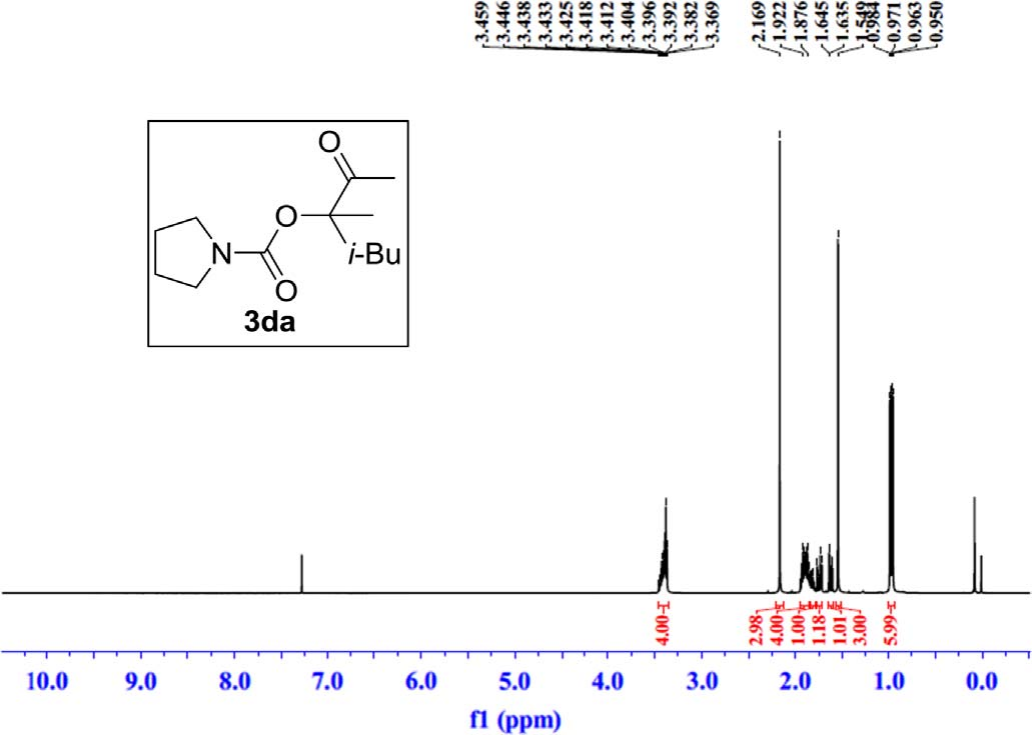
^1^H NMR spectra of **3da**.

**Fig. 12:**
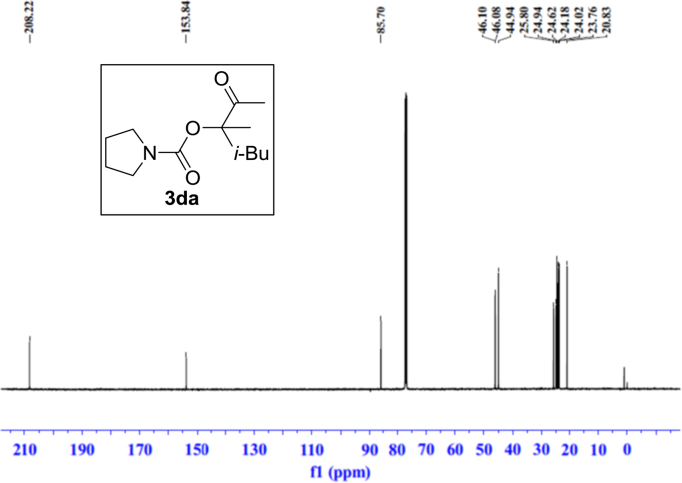
^13^C NMR spectra of **3da**.

**Fig. 13:**
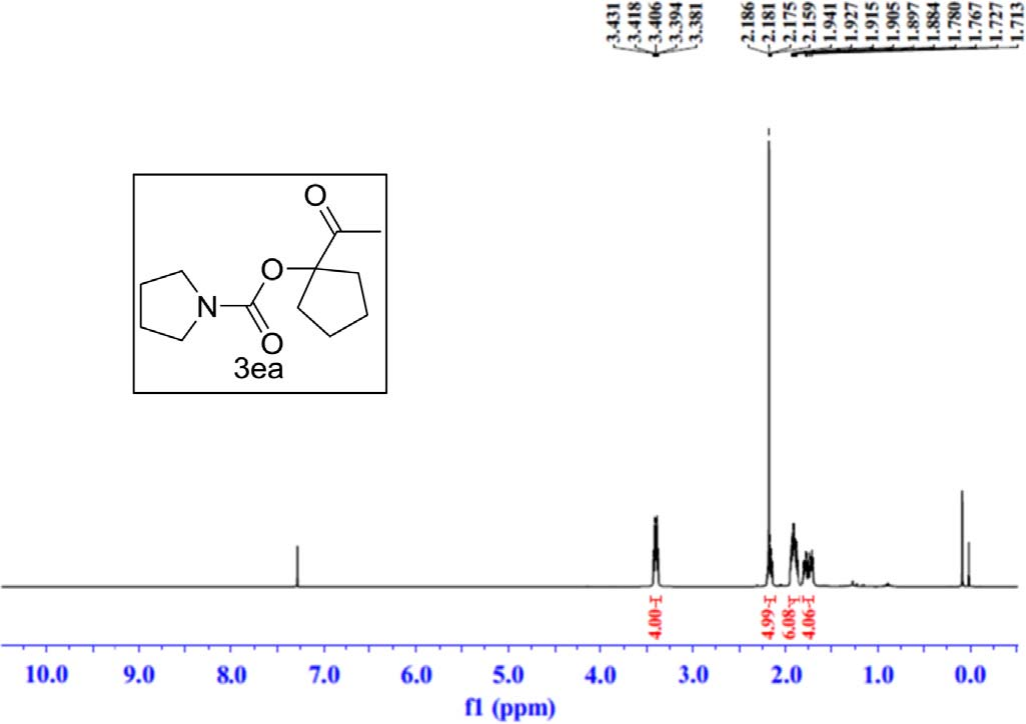
^1^H NMR spectra of **3ea**.

**Fig. 14:**
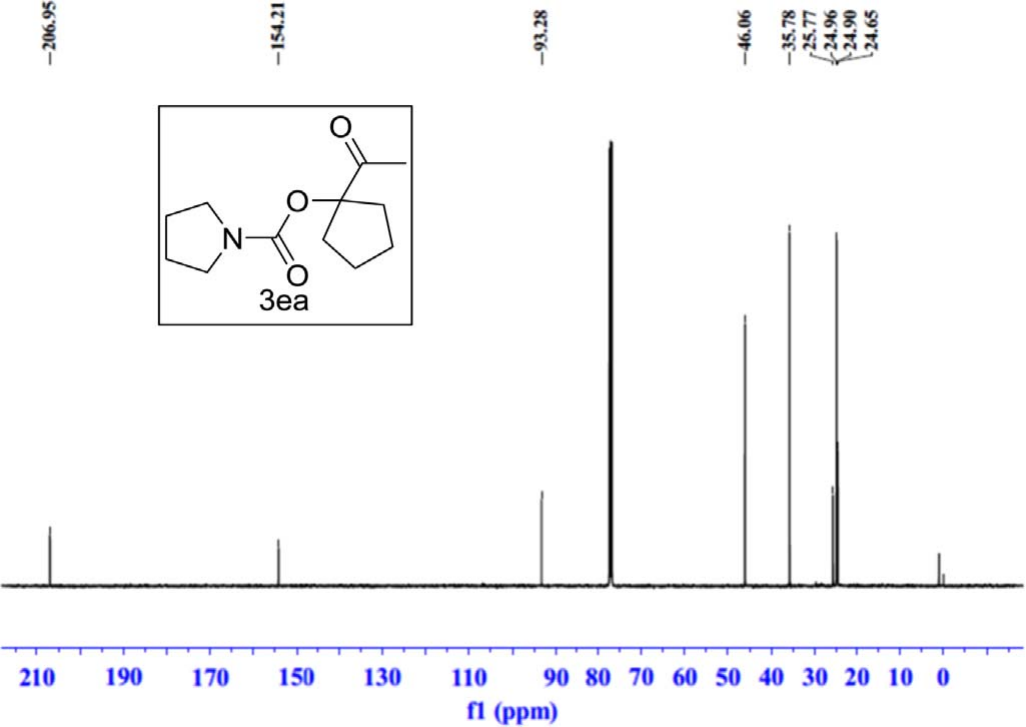
^13^C NMR spectra of **3ea**.

**Fig. 15:**
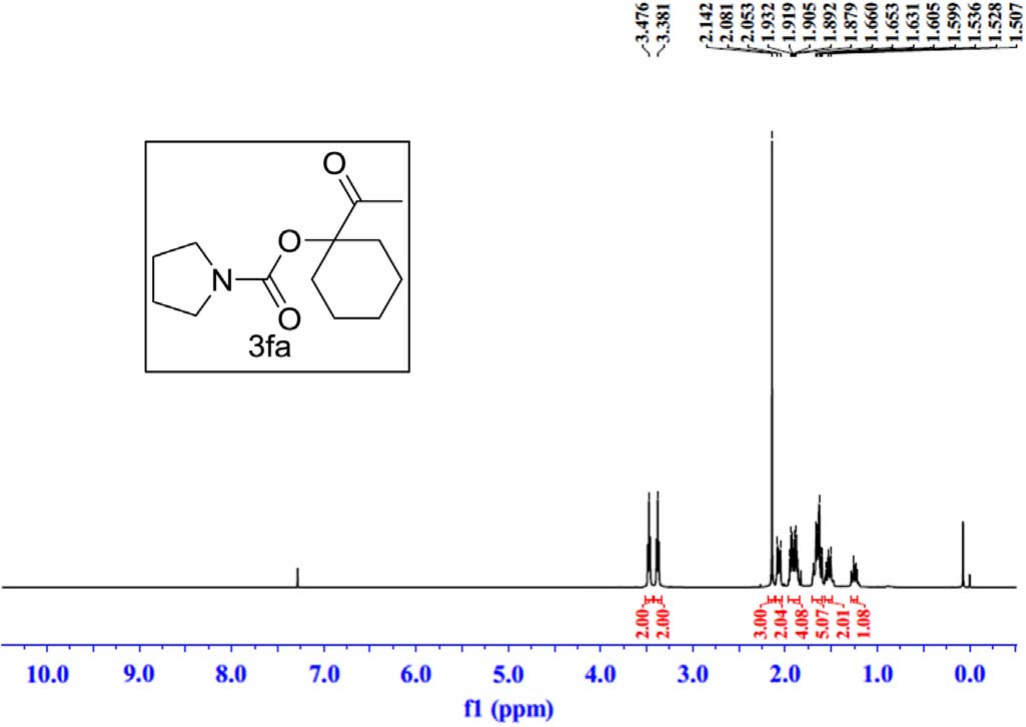
^1^H NMR spectra of **3fa**.

**Fig. 16:**
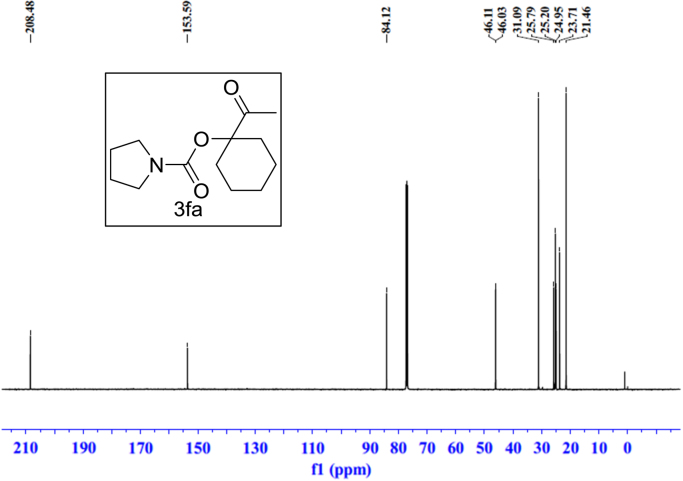
^13^C NMR spectra of **3fa**.

**Fig. 17:**
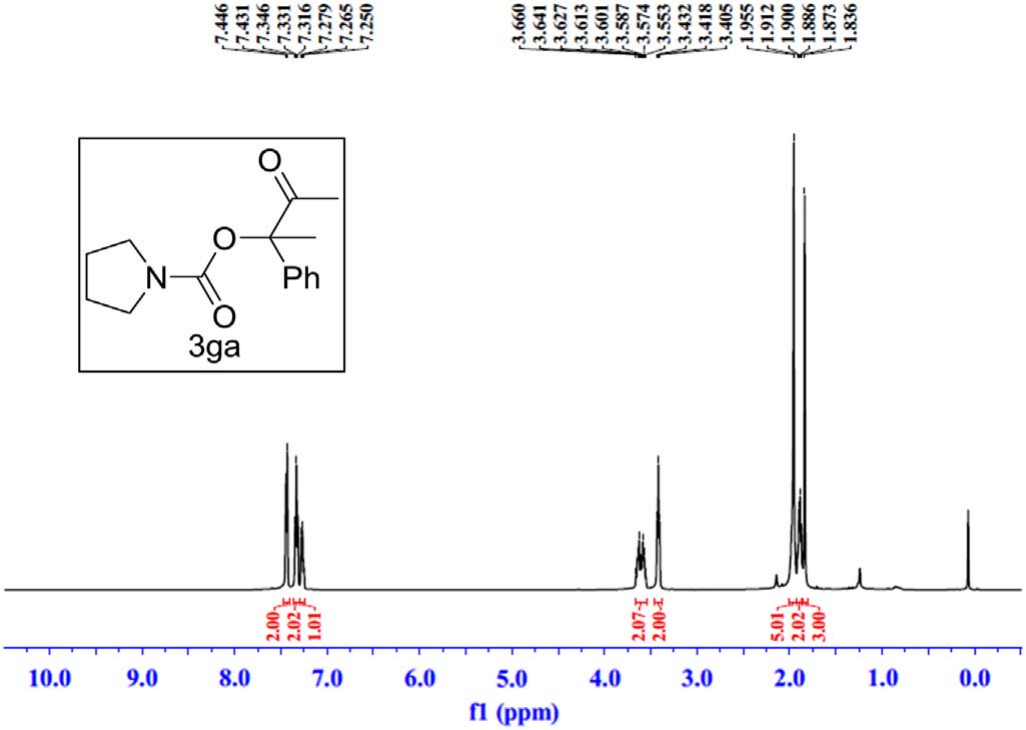
^1^H NMR spectra of **3ga**.

**Fig. 18:**
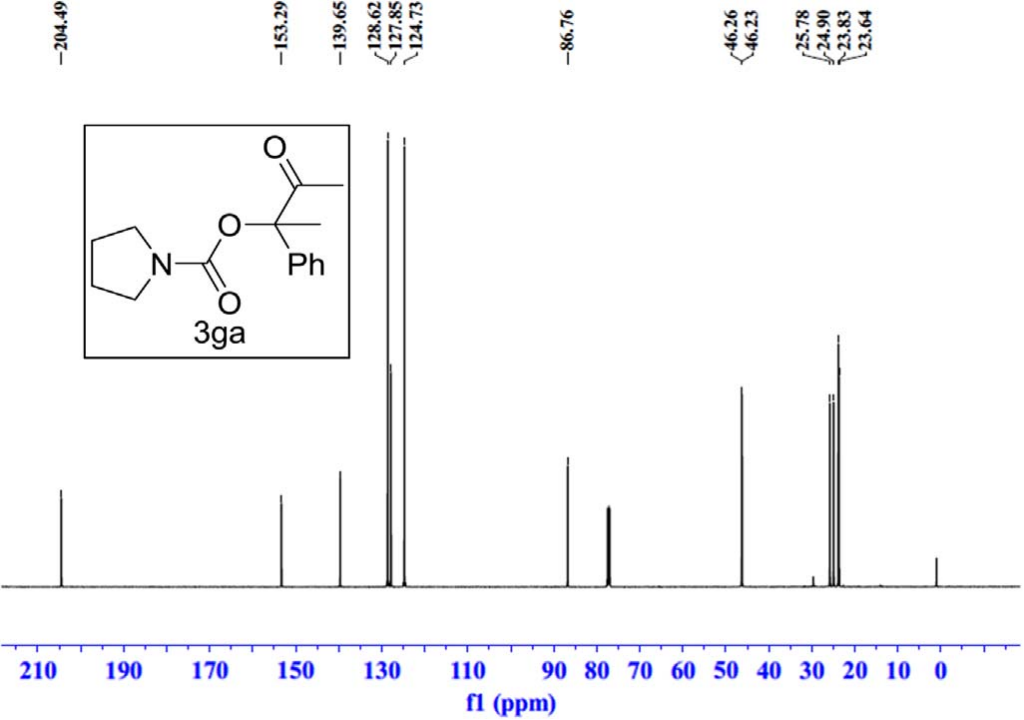
^13^C NMR spectra of **3ga**.

**Fig. 19:**
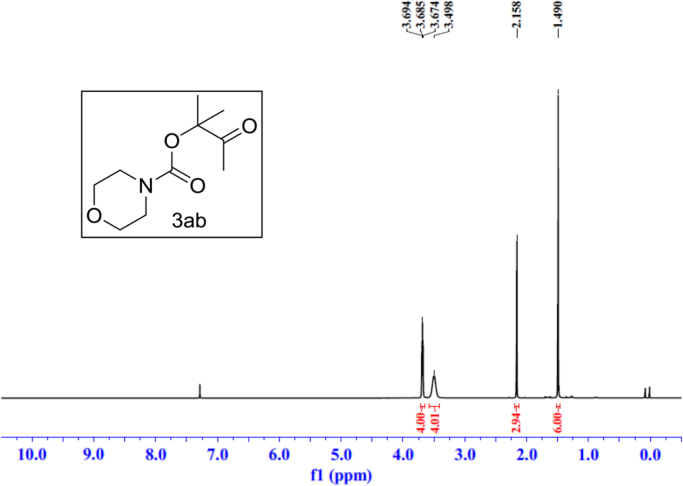
^1^H NMR spectra of **3ab**.

**Fig. 20:**
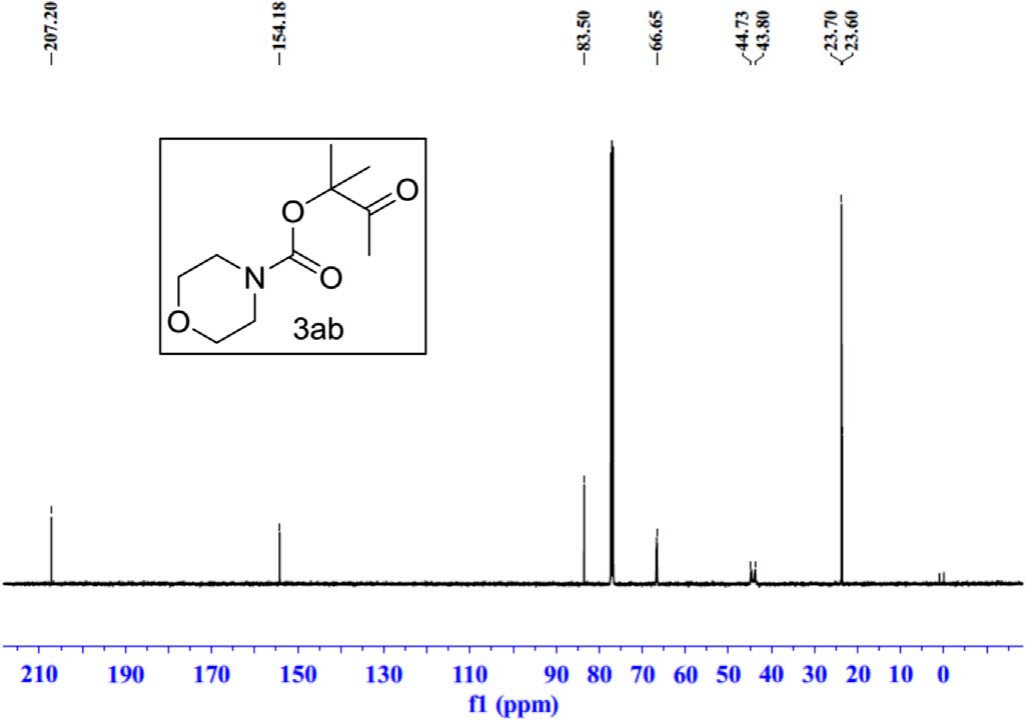
^13^C NMR spectra of **3ab**.

**Fig. 21:**
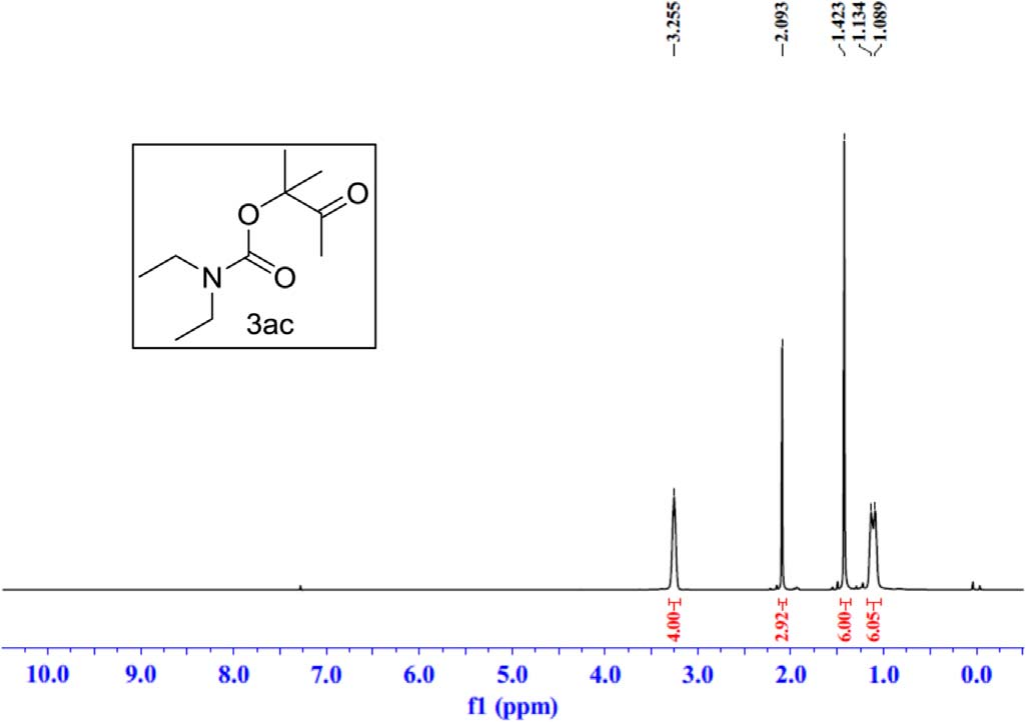
^1^H NMR spectra of **3ac**.

**Fig. 22:**
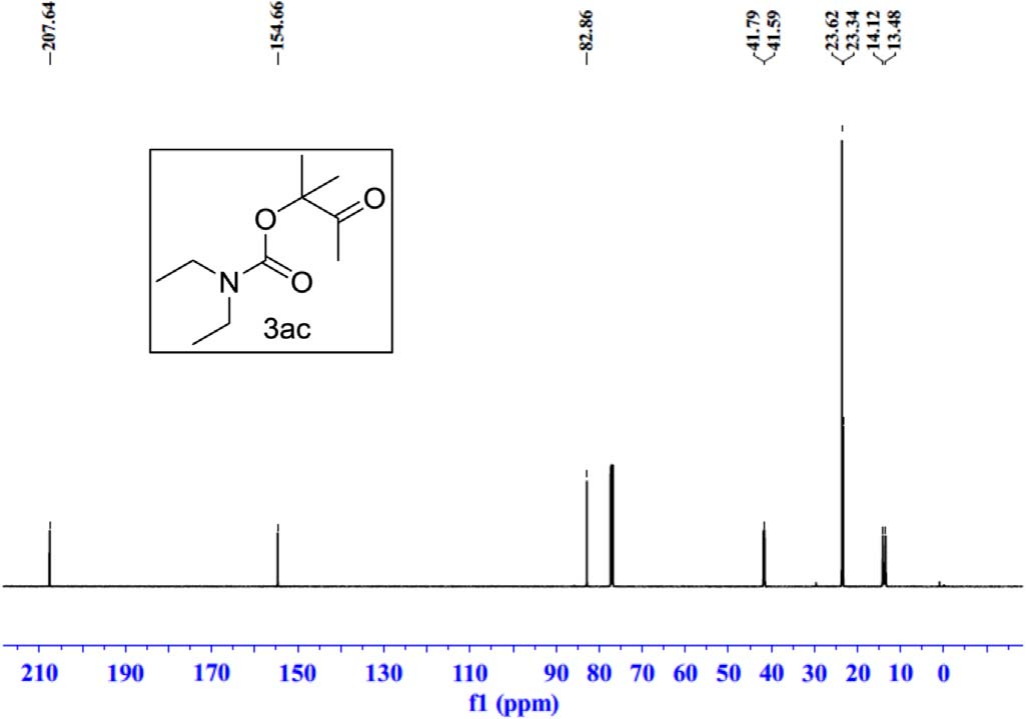
^13^C NMR spectra of **3ac**.

**Fig. 23:**
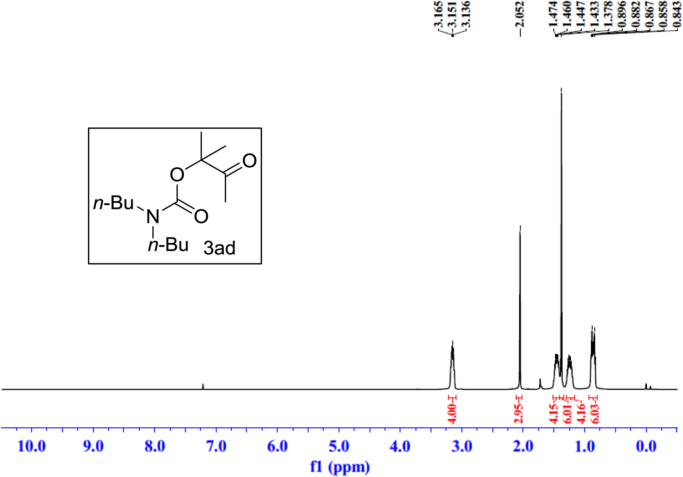
^1^H NMR spectra of **3ad**.

**Fig. 24:**
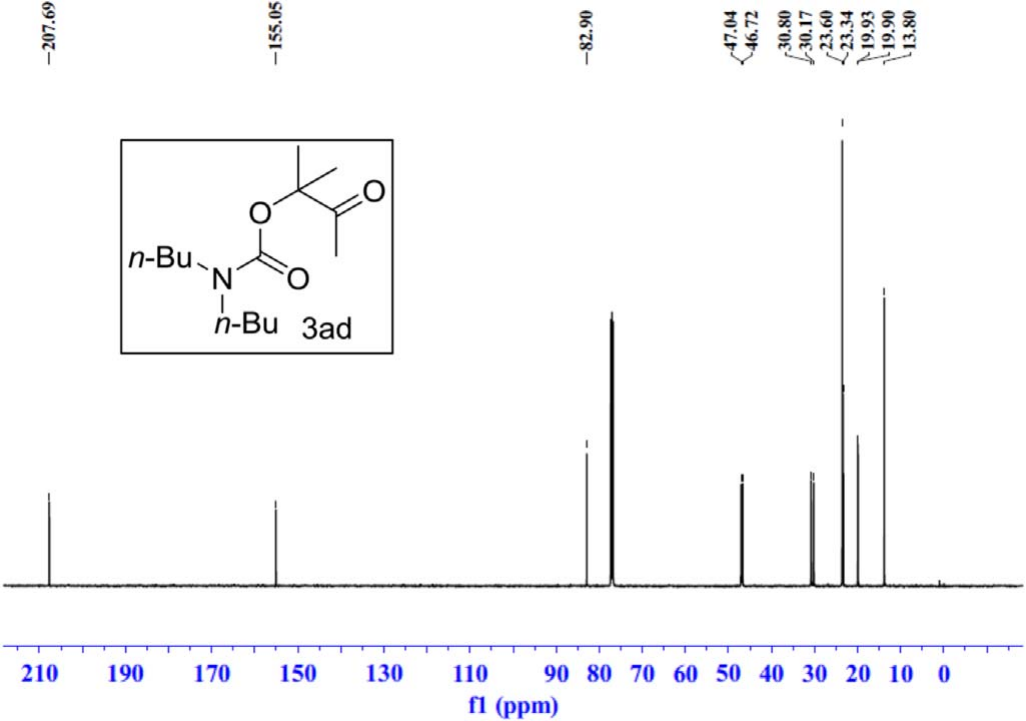
^13^C NMR spectra of **3ad**.

**Fig. 25:**
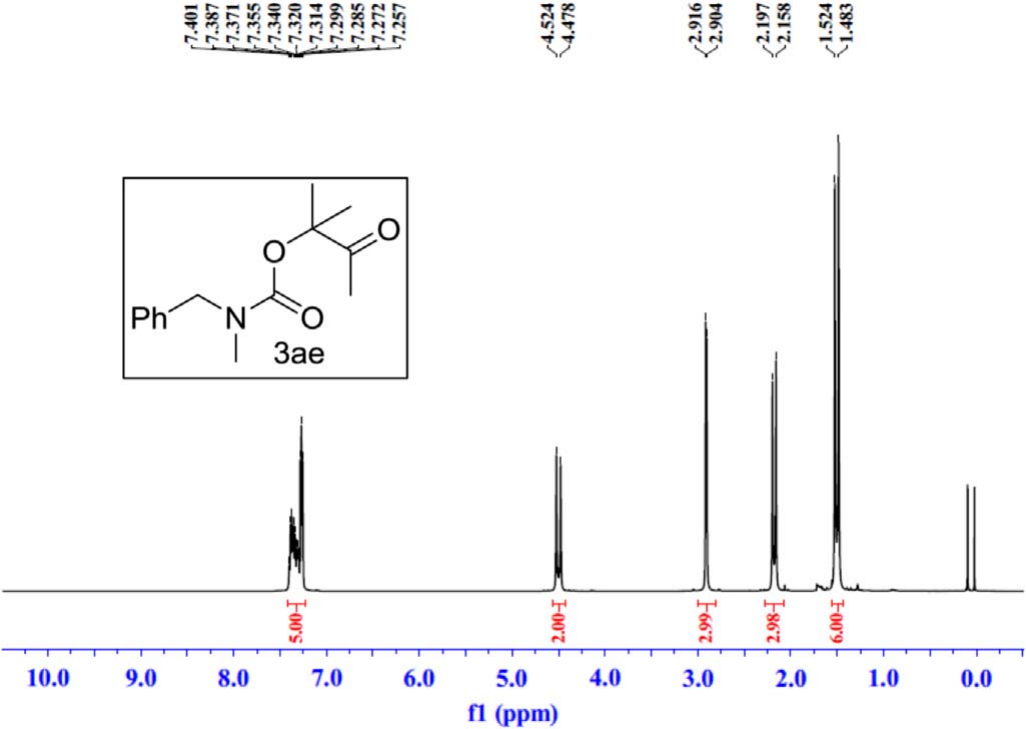
^1^H NMR spectra of **3ae**.

**Fig. 26:**
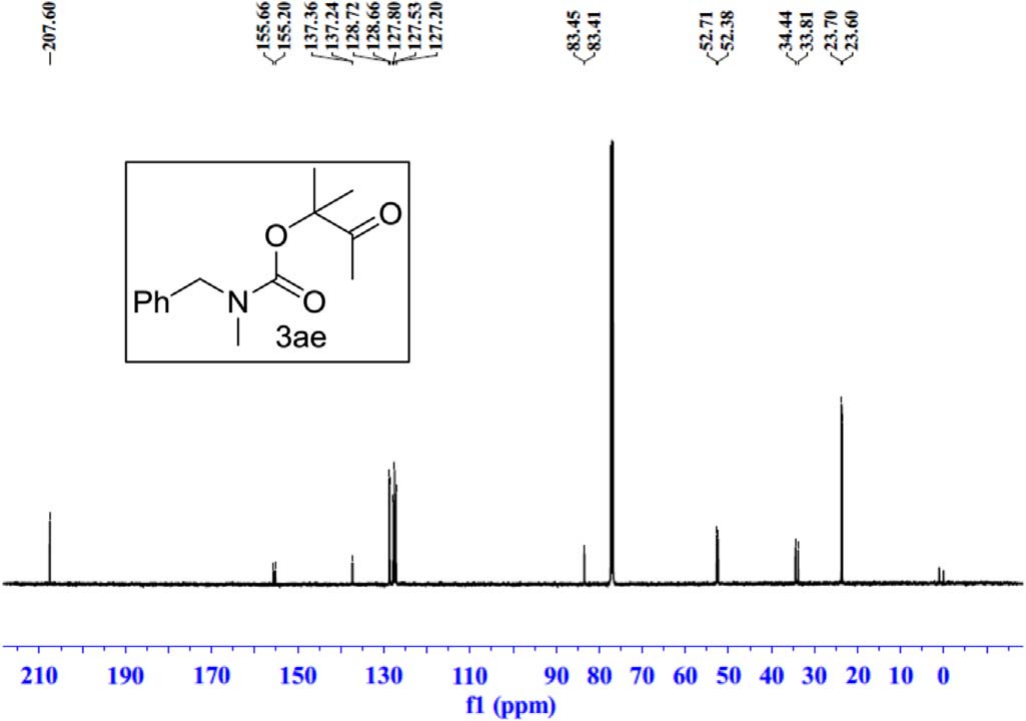
^13^C NMR spectra of **3ae**.

## 2 Experimental design, materials, and methods

### 2.1 General information

All the chemicals utilized were purchased from the commercial companies such as Aladdin, Macklin, Sigma-Aldrich, Alfa, TCI and used immediately without any manipulation. Carbon dioxide (CO_2_) was used in the purity of 99.999% from Wuhan Zhongchunhua And Technology Ltd. ^1^H NMR was performed on a Bruker Avance III HD 500 MHz spectrometers with CDCl_3_ used as the solvent referenced to TMS (*δ* = 0 ppm). ^13^C NMR was performed at 126 MHz in CDCl_3_ and CDCl_3_ (δ = 77.0 ppm) was used as internal reference. Chemical shifts (δ) and coupling constants (J) are given in ppm and hertz (Hz), respectively. HR-MS was recorded on a Bruker Daltonics microTOF-QII mass spectrometer.

### 2.2 The structures of ionic liquids

 See Fig. 1.

### 2.3 The three-component reaction of propargylic alcohols, secondary amines and carbon dioxide

AgBr (0.05 mmol, 1 mol%), [C_2_C_1_im][OAc] (1.25 mmol, 25 mol%), propargylic alcohol (5 mmol) and secondary amine (5 mmol) were added to an Schlenk tube equipped with a stir bar. After the system was purged with СО_2_ three times, the mixture was stirred at 45 °C under 0.1 MPa of CO_2_ for the desired time. When the reaction completed, the mixture was extracted with diethyl ether (3 × 15 mL). The upper layers were collected and dried under vacuum to give the crude products, which could be purified by further column chromatography on silica gel using petroleum ether/ethyl acetate (100:1–20:1) as an eluent. The structures of all target compounds are depicted in Fig. 2.

### 2.4 Effect of the amount of [C_2_C_1_im][OAc] on the coupling reaction

As shown in Table 1, from entry 1 to entry 5, we could observe that the yield increases when the amount of [C_2_C_1_im][OAc] increase, this trend might be explained by the better activation achieved by more amount of IL. When the usage of [C_2_C_1_im][OAc] was increased to 1.25 mmol, a yield of 95% was obtained, further addition of more IL did not help too much to improve the yield.

### 2.5 Effects of temperature and time on the coupling reaction

As shown in Table 2, the yield firstly increased along with the temperature from 25 °C to 45 °C (Table 2, entry 1 vs. 2), and then remained nearly constant when the temperature increased from 45 °C to 65 °C (Table 2, entry 2 vs. 3). The relationship between time and yield was also explored (as shown in Table 3). From 5 h to 7 h, the yield increased rapidly from 70% to 93% (Table 3, entry 1 vs. 2). When the time was extended to 9 h, the yield reached 99% (Table 3, entry 3). Further extending of the reaction time did not show an obvious effect on the yield (Table 3, entry 3 vs. 4).

### 2.6 Procedure for the recycling experiment

The recycling experiments were performed in one-pot stepwise processes to prepare ß-oxopropylcarbamates. In a 25 ml Schlenk tube, AgBr (0.05 mmol, 1 mol%), [C_2_C_1_im][OAc] (1.25 mmol, 25 mol%), propargylic alcohols (5 mmol) were added. The system was purged with СО_2_ three times. Then the mixture was stirred at 45 °C under 0.1 MPa of CO_2_ for 10 h. After that, pyrrolidine (5 mmol) was added into this mixture by a syringe and the system was continued to stir at 45 °C for 1 h. Then, the mixture was extracted with diethyl ether (3 × 15 mL) and the upper layers were combined to obtain the desired products. The lower layer (recovered [C_2_C_1_im][OAc]+AgBr) was reused for the next run after drying under vacuum for 20 min to remove the residual diethyl ether. Moreover, according to ICP the amount of Ag lost to the water phase during the first recycling was 0.74%.

### 2.7 Investigation the probable state of Ag in the ILs

In the mixture of our catalytic system [1], the acetate anion may interact with the proton of the imidazole, generating the free NHC. Then the free NHC may combine with the Ag salt to produce carbene-Ag complexes. In order to explore the possibilities of these complexes, we first synthesized and characterized the pure carbene-Ag complex, then we monitored our catalytic system by NMR and HRMS. Indeed, the results indicated that the complex existed in this system. The experimental details are supplied below.

(1) Synthesis and characterization of the pure carbene-Ag complex
  Ag_2_O (0.25 mmol), 1-ethyl-3-methylimidazolium bromide ([C_2_C_1_im]Br) (0.65 mmol), CH_2_Cl_2_ (5 ml) were added in a 25 ml Schlenk tube, then the mixture was stirred at 45 °C for 3 h. After reaction finished, the mixture was dried under vacuum to remove CH_2_Cl_2_. Then the obtained product was characterized by ^13^C NMR (as shown in Fig. 3a); a peak at *δ* = 181 ppm was observed, which is considered to be the characteristic carbene carbon in the pure carbene-Ag complex.
(2) Detection of the existence of carbene-Ag complex
  We first prepared our catalytic system under the optimal conditions without the addition of the substrate: In a 25 ml Schlenk tube, AgBr (1.25 mmol), [C_2_C_1_im][OAc] (6.45 mmol) were added. The system was purged with СО_2_ three times. Then the mixture was stirred at 45 °C under 0.1 MPa of CO_2_ for 8 h. The reaction mixture was analyzed by ^13^C NMR (Fig. 3b). In Fig. 3b, we also observed the same peak at *δ* = 181 ppm, indicating the existence of a carbene-Ag complex in this system. Furthermore, the other peaks of the pure carbene-Ag complex could also be observed in the ^13^C NMR spectrum of the catalytic system. Moreover, HRMS (Fig. 4) indicated this carbene-Ag complex is an N-heterocyclic bis-carbene silver complex. Two peaks are observed in accordance with the isotopic distribution of silver (HRMS (ESI): m/z calcd. for C_12_H_20_AgN_4_ [M-Br]^+^: 327.07334; Found: 327.07365).

## 3 The characterization of products

All the NMR and HRMS data for the target products are supplied in Supplementary material.

### 3.1 NMR spectral of the products

 See Fig. 6, Fig. 7, Fig. 8, Fig. 9, Fig. 10, Fig. 11, Fig. 12, Fig. 13, Fig. 14, Fig. 15, Fig. 16, Fig. 17, Fig. 18, Fig. 19, Fig. 20, Fig. 21, Fig. 22, Fig. 23, Fig. 24, Fig. 25, Fig. 26
