# Supplementary material for: Data for the synthesis of β-oxopropylcarbamates from propargylic alcohols, secondary amines and CO2 catalyzed by a recyclable AgBr/ionic liquid system under ambient pressure
Source: Data Brief. 2018 Sep 5;20:1378–91. doi: 10.1016/j.dib.2018.08.183 (PMC6148840; doi:10.1016/j.dib.2018.08.183)
Supplement: Supplementary file 1 — Supplementary material. [file mmc1.docx]

**Conflict of Interest**

I am authorized on behalf of all the authors of this manuscript to confirm that no author has any conflict of interest to disclose
