# Supplementary material for: Data for the synthesis of β-oxopropylcarbamates from propargylic alcohols, secondary amines and CO2 catalyzed by a recyclable AgBr/ionic liquid system under ambient pressure
Source: Data Brief. 2018 Sep 5;20:1378–91. doi: 10.1016/j.dib.2018.08.183 (PMC6148840; doi:10.1016/j.dib.2018.08.183)
Supplement: Supplementary file 2 — Supplementary material. [file mmc2.docx]

**Supplementary material**

**Title**: Data for the synthesis of β-oxopropylcarbamates from propargylic alcohols, secondary amines and CO_2_ catalyzed by a recyclable AgBr/ionic liquid system under ambient pressure

**Authors**: Dandan Song^a,b^, Di Li^a,b^, Xuan Xiao^a,b^, Cheng Chen^a^, Somboon Chaemchuen^a^, Ye Yuan^a,^*, Francis Verpoort^a,b,c,d,^*

**Affiliations**:

*^a^* State Key Laboratory of Advanced Technology for Materials Synthesis and Processing, Wuhan University of Technology, Wuhan 430070 (P.R. China)

*^b^* School of Material Science and Engineering, Wuhan University of Technology, Wuhan 430070 (P.R. China)

*^c^* National Research Tomsk Polytechnic University, Lenin Avenue 30, Tomsk 634050 (Russian Federation)

*^d^* Global Campus Songdo, Ghent University, 119 Songdomunhwa-Ro, Yeonsu-Gu, Incheon (Korea)

**Contact email**: fyyuanye@whut.edu.cn, [francis.verpoort@ghent.ac.kr](mailto:francis.verpoort@ghent.ac.kr).

1. **The characterization of products**

 **3aa**

*Colorless oil. ^1^H NMR (500 MHz, CDCl_3_) δ 3.40-3.38 (m, 4H, H1, H4), 2.17 (s, 3H, H10), 1.96-1.82 (m, 4H, H2-H3), 1.48 (s, 6H, H7-H8). ^13^C NMR (126 MHz, CDCl_3_) δ 208.0 (C9), 153.9 (C5), 82.9 (C6), 46.1, 46.0 (C1, C4), 25.8 (C2, C3), 24.9 (C10), 23.8, 23.7 (C7, C8). HRMS (ESI): m/z calcd. for C_10_H_18_NO_3_ [M+H]^+^: 200.12812; Found: 200.12814.*

*The NMR spectroscopic data matched those reported in the literature [1].*

**3ba**

*Straw yellow oil. ^1^H NMR (500 MHz, CDCl_3_) δ 3.41-3.37 (m, 4H, H1, H4), 2.15 (s, 3H, H11), 1.93-1.69 (m, 6H, H2-H3, H8), 1.48 (s, 3H, H7), 0.90 (t, J = 7.8 HZ, 3H, H9). ^13^C NMR (126 MHz, CDCl_3_) δ 208.1 (C10), 153.8 (C5), 85.5 (C6), 46.0 (C1, C4), 29.7 (C8), 25.8 (C11), 24.9, 24.3 (C2, C3), 20.1 (C7), 7.6 (C9). HRMS (ESI): m/z calcd. for C_11_H_20_NO_3_ [M+H]^+^: 214.14377; Found: 214.14379.*

**3ca**

*Straw yellow oil****.*** *^1^H NMR (500 MHz, CDCl_3_) δ 3.41-3.36 (m, 4H, H1, H4), 2.14 (s, 3H, H12), 2.02-1.87 (m, 8H, H2-H3, H7-H8), 0.79 (t, J = 7.5 Hz, 6H, H9-H10). ^13^C NMR (126 MHz, CDCl_3_) δ 208.4 (C11), 153.6 (C5), 88.5 (C6), 46.04, 46.02 (C1, C4), 25.8 (C12), 25.3, 25.2, 24.9 (C2, C3, C7, C8), 7.4 (C9, C10). HRMS (ESI): m/z calcd. for C_12_H_22_NO_3_ [M+H]^+^: 228.15942; Found: 228.15951.*

**3da**

*Straw yellow oil. 1H NMR (500 MHz, CDCl3) δ 3.46-3.37 (m, 4H, H1, H4), 2.17 (s, 3H, H13), 1.92-1.72 (m, 6H, H2-H3, H8), 1.65-1.60 (m, 1H, H9), 1.55 (s, 3H, H7), 0.98-0.95 (m, 6H, H10-H11). 13C NMR (126 MHz, CDCl3) δ 208.2 (C12), 153.8 (C5), 85.7 (C6), 46.10, 46.08 (C1, C4), 44.94 (C8), 25.8 (C13), 24.9, 24.6 (C2, C3), 24.2, 24.0 (C10, C11), 23.8 (C9), 20.8 (C7). HRMS (ESI): m/z calcd. for C13H24NO3 [M+H]+: 242.17507; Found: 242.17517.*

**3ea**

*Straw yellow oil. ^1^H NMR (500 MHz, CDCl_3_) δ 3.43-3.38 (m, 4H, H1, H4), 2.20-2.15 (m, 5H, H7a, H10a, H12), 1.95-1.87 (m, 6H, H2, H3, H7b, H10b), 1.80-1.70 (m, 4H, H8-H9). ^13^C NMR (126 MHz, CDCl_3_) δ 207.0 (C11), 154.2 (C5), 93.3 (C6), 46.1 (C1, C4), 35.8 (C7, C10), 25.8 (C12), 25.0, 24.9 (C2, C3), 24.7 (C8, C9). HRMS (ESI): m/z calcd. for C_12_H_20_NO_3_ [M+H]^+^: 226.14377; Found: 226.14385.*

**3fa**

*Colorless solid. ^1^H NMR (500 MHz, CDCl_3_) δ 3.48 (t, J = 6.7 Hz, 2H, H1 or H4), 3.38 (t, J = 6.7 Hz, 2H, H4 or H1), 2.14 (s, 3H, H13), 2.08-2.05 (m, J = 13.8 Hz, 2H, H7a, H11a), 1.95-1.87 (m, 4H, H2-H3), 1.69-1.60 (m, 5H, H7b-H11b), 1.55-1.50 (m, 2H, H8a, H10a), 1.29-1.22 (m, 1H, H9a). ^13^C NMR (126 MHz, CDCl_3_) δ 208.5 (C12), 153.6 (C5), 84.1 (C6), 46.1, 46.0 (C1, C4), 31.1 (C7, C11), 25.8 (C13), 25.2, 25.0 (C2, C3), 23.7 (C8, C10), 21.5 (C9). HRMS (ESI): m/z calcd. for C_13_H_22_NO_3_ [M+H]^+^: 240.15942; Found: 240.15950.*

*The NMR spectroscopic data matched those reported in the literature [2].*

**3ga**

*White solid. ^1^H NMR (500 MHz, CDCl_3_) δ 7.45-7.43 (m, 2H, H9, H11), 7.35-7.32 (m, 2H, H8, H12), 7.28-7.25 (m, 1H, H10), 3.66-3.55 (m, 2H, H1 or H4), 3.42 (t, J = 6.7 Hz, 2H, H4 or H1), 1.95-1.87 (m, 7H, H2-H3, H15), 1.84 (s, 3H, H13). ^13^C NMR (126 MHz, CDCl_3_) δ 204.5 (C14), 153.3 (C5), 139.7 (C7), 128.6 (C9, C11), 127.9 (C8, C12), 124.7 (C10), 86.8 (C6), 46.3, 46.2 (C1, C4), 25.8, 24.9, 23.8, 23.6 (C2, C3, C13, C15). HRMS (ESI): m/z calcd. for C_15_H_20_NO_3_ [M+H]^+^: 262.14377; Found: 262.14383.*

*The NMR spectroscopic data matched those reported in the literature [1].*

**3ab**

*Orange solid. ^1^H NMR (500 MHz, CDCl_3_) δ 3.69 (t, J = 5.0 MHz, 4H, H1, H4), 3.57-3.45 (m, 4H, H2, H3), 2.16 (s, 3H, H10), 1.49 (s, 6H, H7, H8). ^13^C NMR (126 MHz, CDCl_3_) δ 207.2 (C9), 154.2 (C5), 83.5 (C6), 66.7 (C2, C3), 44.7, 43.8 (C1, C4), 23.7, 23.6 (C7, C8, C10). HRMS (ESI): m/z calcd. for C_10_H_18_NO_4_ [M+H]^+^: 216.12303; Found: 216.12314.*

**3ac**

*Straw yellow oil. ^1^H NMR (500 MHz, CDCl_3_) δ 3.31-3.18 (m, 4H, H1, H2), 2.09 (s, 3H, H10), 1.42 (s, 6H, H7, H8), 1.13-1.08 (m, 6H, H3, H4). ^13^C NMR (126 MHz, CDCl_3_) δ 207.6 (C9), 154.7 (C5), 82.9 (C6), 41.8, 41.6 (C1, C2), 23.6, 23.3 (C7, C8, C10), 14.1, 13.5 (C3, C4). HRMS (ESI): m/z calcd. for C_10_H_20_NO_3_ [M+H]^+^:202.14377; Found: 202.14383.*

*The NMR spectroscopic data matched those reported in the literature [3].*

**3ad**

*Yellow oil. ^1^H NMR (500 MHz, CDCl_3_) δ 3.15 (t, J = 7.2 MHz, 4H, H1-H2), 2.05 (s, 3H, H14), 1.49-1.41 (m, 4H, H3-H4), 1.38 (s, 6H, H11-H12), 1.29-1.20 (m, 4H, H5-H6), 0.89-0.83 (m, 6H, H7-H8). ^13^C NMR (126 MHz, CDCl_3_) δ 207.7 (C13), 155.1 (C9), 82.9 (C10), 47.04, 46.72 (C1, C2), 30.8, 30.2 (C3, C4), 23.6, 23.3 (C11, C12, C14), 19.93, 19.90 (C5, C6), 13.8 (C7, C8). HRMS (ESI): m/z calcd. for C_14_H_28_NO_3_ [M+H]^+^: 258.20637; Found: 258.20659.*

*The NMR spectroscopic data matched those reported in the literature [4].*

**3ae**

*Straw yellow oil. ^1^H NMR (500 MHz, CDCl_3_) 1:1.07 mixture of rotamers. Major rotamer: δ 7.40-7.26 (m, 5H, H2-H6), 4.52 (s, 2H, H7), 2.92 (s, 3H, H8), 2.16 (s, 3H, H14), 1.48 (s, 6H, H11-H12). Minor rotamer: δ 7.40-7.26 (m, 5H, H2-H6), 4.48 (s, 2H, H7), 2.90 (s, 3H, H8), 2.20 (s, 3H, H14), 1.52 (s, 6H, H11-H12). ^13^C NMR (126 MHz, CDCl_3_) 1:1.07 mixture of rotamers. Major rotamer: δ 207.6 (C13), 155.7 (C9), 137.4 (C1), 128.7 (C2, C6), 127.8, 127.5 (C3, C5), 127.2 (C4), 83.5 (C10), 52.7 (C7), 34.4 (C8), 23.7, 23.6 (C11, C12, C14). Minor rotamer: δ 207.6 (C13), 155.2 (C9), 137.2 (C1), 128.7 (C2, C6), 127.8, 127.5 (C3, C5), 127.2 (C4), 83.4 (C10), 52.4 (C7), 33.8 (C8), 23.7, 23.6 (C11, C12, C14). HRMS (ESI): m/z calcd. for C_14_H_20_NO_3_ [M+H]^+^: 250.14377; Found: 250.14397.*

*The NMR spectroscopic data matched those reported in the literature [3].*

**References**

*[1] N. D. Ca, B. Gabriele, G. Ruffolo, L. Veltri, T. Zanetta, M. Costa, Effective guanidine-catalyzed synthesis of carbonate and carbamate derivatives from propargyl alcohols in supercritical carbon dioxide. Adv. Synth. Catal.* ***2011,*** *353 (1), 133-146.*

*[2] Q. W. Song, B. Yu, X. D. Li, R. Ma, Z. F. Diao, R. G. Li, W. Li, L. N. He, Efficient chemical fixation of CO_2_ promoted by a bifunctional Ag_2_WO_4_/Ph_3_P system. Green Chem.* ***2014,*** *16 (3), 1633-1638.*

*[3]* *X. D. Li, X. D. Lang, Q. W. Song, Y. K. Guo, L. N. He, Cu(I)-catalyzed three-component reaction of propargylic alcohol, secondary amines and atmospheric CO_2_. Chin. J. Org. Chem* ***2016,*** *36, 744-751.*

*[4] Q. W. Song, Z. H. Zhou, H. Yin, L. N. He, Silver(I)-catalyzed synthesis of β-oxopropylcarbamates from propargylic alcohols and CO_2_ surrogate: A gas-free process. ChemSusChem* ***2015,*** *8 (23), 3967-3972.*
